# Comment on “Solar maculopathy secondary to sunlight exposure reflected from the screen of mobile devices: two case reports”

**DOI:** 10.1186/s13256-023-04009-6

**Published:** 2023-06-14

**Authors:** Bruno Piccoli, Dino Pisaniello, Emanuela Bonci, Silvano Orsini, Roberto Di Censi

**Affiliations:** 1 Department of Biomedicine, Section of Occupational Health, University of Tor Vergata, Rome, Italy; 2 School of Public Health, The University of Adelaide, Adelaide, 5005 Australia

Dear Sir,

We read with great attention the article “Solar maculopathy secondary to sunlight exposure reflected from the screen of mobile devices: two case reports” by Joaquín Marticorena *et al*. [1]

Based on the data reported in the article, there is no evidence that the tablet and the mobile phone used by the two cases described played a significant role in the development of a bilateral maculopathy.

Certainly, the two patients considered could have been also exposed to a huge amount of blue light originating from the environment (terrace of a ski centre and beach), while they were looking around. It is unlikely that the two patients considered would have been reading from screens with high luminance for extended periods (i.e. the characters would be washed out, with insufficient contrast). It is likely that their gaze included reflected blue light from other sources.

Blue light exposure comes from the ambient outdoor reflected blue light (extended occupational visual field) [2], as well as specular light from the screen, of which no reflection properties are described in the paper. We believe both conditions should have been specifically considered and analyzed by the authors, but no data are reported in the article in this regard, i.e. specular luminance measurement from the screen in addition to specular luminance from walls, snow, water, etc. Furthermore, no measurements were undertaken and no laboratory or outdoor simulations of the scenarios mentioned, where spectroradiometric measurements are required to understand risk according to ICNIRP [3].

Importantly, reflected light which could have caused the maculopathy cannot be assessed in terms of luminance, as stated on page 3 of the article. Luminance is a photometric term and not appropriate for radiometry (luminance does not determine the light spectra, as radiometric measures do). In addition, radiance is essential to comply with the Blue Light exposure criterion proposed by ICNIRP (100 J/cm^2^-sr over a total viewing time of 167 min in a day) and fully adopted by ACGIH as a TLV [4].

To present knowledge, only Blue Light (380–550 nm) can cause a photo-maculopathy (photoretinitis), while UV-B light (315–280 nm), being largely blocked by the lens cannot, as the authors have reported on page 3 of the article.

The author’s conclusion that specular blue light from the screen is the cause of the maculopathy is not justified, based on the major confounder of ambient reflected blue light in ski resorts, outdoor fishing and beach activity [5]. The conclusion, which is not consistent with the Directive of the European Parliament and of the Council on the Minimum Health and Safety Requirements Regarding the Exposure of Workers to Risks Arising from Physical Agents (Artificial Optical Radiation), can also be misconstrued by advocates of employment conditions, as well as occupational health and safety advisers.

Regards

## Data Availability

Reported in the bibliography.
